# A Review of Susceptibility Factors for Colibactin-Associated Colorectal Cancer in African Populations

**DOI:** 10.7759/cureus.96473

**Published:** 2025-11-10

**Authors:** Olurotimi J Badero, Onyinye I Ikedionwu, Victor Ajayi, Abayomi V Jemiseye

**Affiliations:** 1 Interventional Cardiology, Iwosan Lagoon Hospital, Lagos, NGA; 2 Interventional Cardiology, Cardiac, Renal, and Vascular Associates P.C., Jackson, USA; 3 Internal Medicine, College of Medicine, University of Lagos, Idi-Araba, NGA; 4 Medicine, Lagos State University Teaching Hospital, Ikeja, NGA; 5 Medicine and Surgery, College of Medicine, University of Lagos, Idi-Araba, NGA

**Keywords:** african population, carcinogenesis, colibactin, colorectal cancer, genetic susceptibility

## Abstract

Colorectal cancer (CRC) is a significant public health concern in Africa. The rising incidence has been attributed to dietary and lifestyle modifications. Gut microbiota, particularly the bacterium *Escherichia coli* and its colibactin toxin, have been implicated in the development of CRC. Due to their ability to induce genomic instability and DNA damage in colonic epithelial cells, colibactin-producing *E. coli* strains have been linked to CRC. Determining high-risk individuals and creating focused prevention and treatment plans requires an understanding of the genetic susceptibility to colibactin-induced colorectal cancer among African communities. A scoping review was conducted aimed at synthesizing current knowledge on the mutagenic properties of polyketide synthase-positive *E. coli* (pks+ *E. coli*) and exploring genomic landscape and population-specific factors that may increase CRC susceptibility in African communities. This review found that African populations' genetic vulnerability to colibactin-induced CRC exhibits different genetic profiles than those of European populations, as the Kirsten Rat Sarcoma Viral Oncogene Homolog (KRAS) and Wingless-related Integration Site (WNT) signaling pathway mutations are highly implicated among individuals of African descent. Additionally, other co-existing factors such as Lynch syndrome, Inflammatory bowel disease, parasitic infestation, and dietary changes influence the incidence rates of CRC among this population. Consequently, this information may help reduce the rising rate of CRC in African populations through lifestyle modification and chemotherapy choices. To fully understand the complex interactions among environmental variables, genetic vulnerability, and colibactin-induced colorectal carcinogenesis in African populations, further investigation is necessary.

## Introduction and background

Colorectal cancer (CRC) represents a major global health challenge, with incidence rates that show considerable geographic and population-based variation [1]. Over the years, an increasing prevalence of CRC has been observed across Africa, with the recent age-standardized incidence rate (ASIR) at 8.4 per 100,000 [2], which has been linked to complex interactions between genetic susceptibility, environmental exposures, and lifestyle factors [3].

Gut microbiota are increasingly recognized as a key factor in CRC pathogenesis, with colibactin-producing polyketide synthase-positive (pks+) *Escherichia coli* strains implicated as significant contributors to tumor development [4]. In CRC patients, these strains occur more frequently than in healthy cohorts [5], with their genotoxic product, colibactin, inducing DNA double-strand breaks and subsequent genomic instability. This molecular damage can disrupt tumor suppressor genes, activate oncogenes, and drive oncogenic transformation, particularly in genetically susceptible hosts [6].

The molecular pathogenesis of CRC involves acquired genetic alterations in key pathways regulating DNA repair, cell proliferation, and apoptosis. Specific genetic factors may contribute to risk in African populations, including reported variants in mismatch repair genes such as MutL Homolog-1 (*MLH1*), MutS Homolog-2 (*MSH2*), and oncogenic drivers Adenomatous Polyposis Coli (*APC*), Kirsten Rat Sarcoma Viral Oncogene Homolog (*KRAS*), Tumor Protein P53 (*TP53*) [7,8]. Mutations in *APC*, for instance, can lead to β-catenin accumulation and intrinsic activation of the wingless-related integration site (WNT) signaling pathway, promoting uncontrolled cellular proliferation. Dietary and environmental factors, such as high consumption of red meat and low fiber intake, further compound the risk [9]. Additional evidence regarding the role of microbial contributors was found in a study of Pakistani CRC patients, which reported a high prevalence of colibactin-producing *E. coli* within the B2 phylogroup [10]. In contrast, frequent *KRAS* mutations and microsatellite instability-high (MSI-H) tumors were identified in South African Black patients [7], suggesting distinct etiologic subtypes. Additional support for microbiota-mediated carcinogenesis comes from studies linking antibiotic use to increased CRC risk, possibly through disruption of microbial homeostasis [3].

Despite these insights, the genetic susceptibility of African populations to colibactin-associated CRC remains inadequately characterized. This review aims to synthesize current knowledge on the mutagenic properties of pks+ *E. coli* and explore population-specific factors that may increase CRC susceptibility in African communities.

## Review

Methods

A rigorous and comprehensive search was done using PubMed, Google Scholar, Wiley Online Library, Science Direct, and SpringerLink databases. Our search was tailored and limited to English-language articles published between 2010 and 2025, with search words such as "Colorectal Cancer in Africa", "Risk factor for colorectal cancer in Africans", "Colibactin colorectal cancer", "Genetic risk of colibactin colorectal cancer in Africans and Black Americans", and "risk factors of Africans to colibactin colorectal cancer" were used.

Eligibility Criteria

Inclusion criteria included: (i) English-language articles that were published from 2010 to 2025, (ii) Studies that were performed or carried out on humans, (iii) Studies that were published in a peer-reviewed journal, (iv) Studies that focused on the genetic and other risk factors of colibactin CRC in African populations.

Exclusion criteria included: (i) Studies focused on animals, (ii) Studies not focused on CRC or colibactin-associated CRC, (iii) non-peer-reviewed articles, and (iv) Studies not published in English. Studies published before 2010 were excluded as early research on pks⁺ *E. coli *was largely mechanistic, lacking robust epidemiological data, standardized detection methods, or validated human mutational signatures linking it to CRC.

Screening

**Figure 1:**
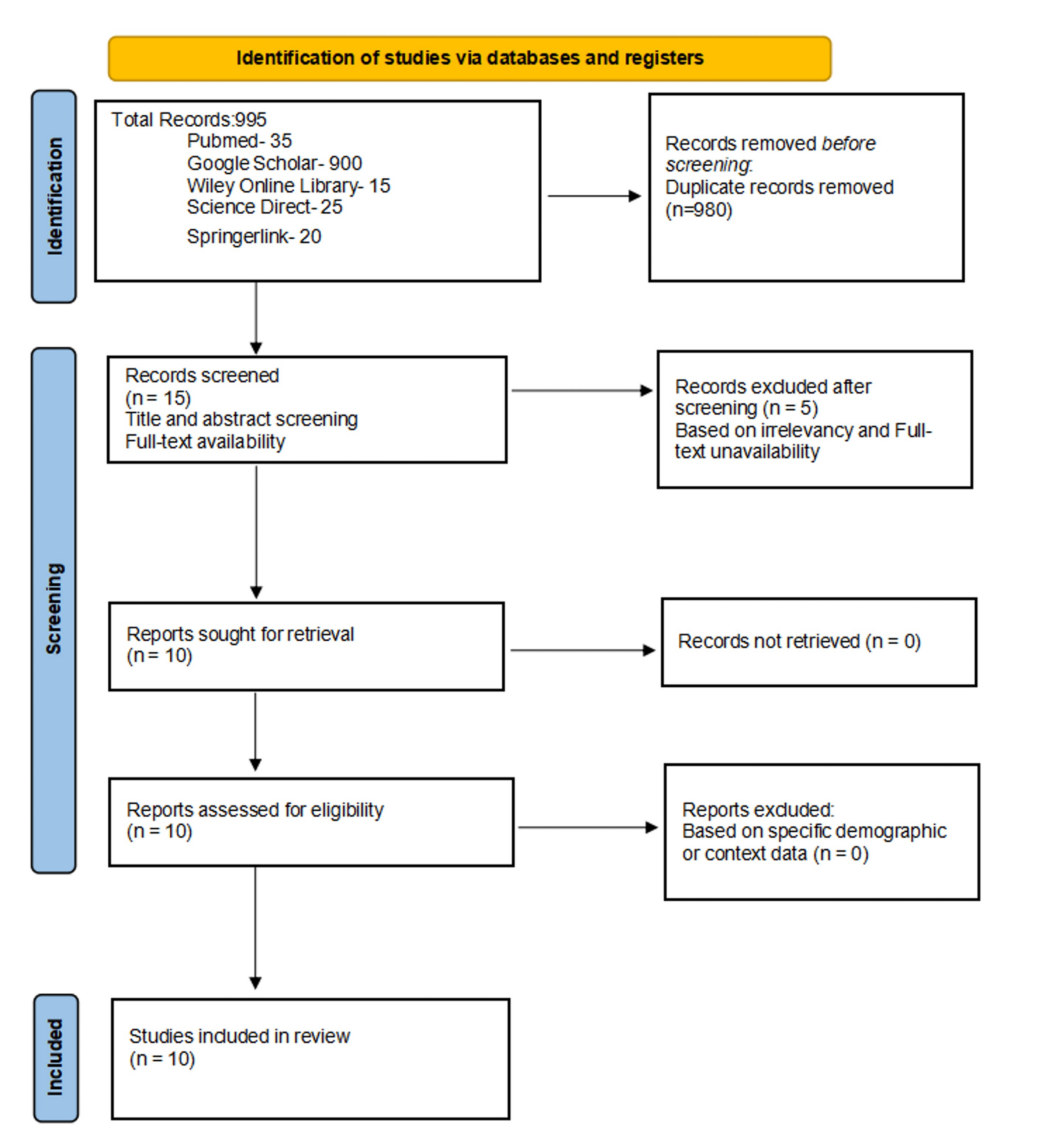
PRISMA flow diagram of the selection process based on the study’s inclusion and exclusion criteria PRISMA: Preferred Reporting Items for Systematic Reviews and Meta-Analyses

The screening process was done in three stages based on title, abstract, and full article screening. Two Independent reviewers discussed discrepancies in titles and full-text reviews, and conflicts were reconciled by a third reviewer or by consensus, as needed, until a conclusion was reached. The studies and papers were systematically analyzed, each undergoing a thorough qualitative assessment, as depicted in Figure 1.

Results

A total of 995 papers were identified; 35 were from PubMed (last searched June 10, 2025), 900 were from Google Scholar (July 8, 2025), 15 from Wiley Online Library (July 12, 2025), 25 from Science Direct (July 15, 2025), and 20 from SpringerLink (July 16, 2025). To minimize duplication and bias, all records were de-duplicated across databases using reference management software before screening, and inclusion decisions were based strictly on predefined eligibility criteria.

The exclusions included 980 duplicated publications. A total of five publications were excluded during the screening process, leaving 10 for retrieval and manual screening. These publications were manually screened based on availability, article type, and title, and were found to satisfy the eligibility criteria and were used in this article.

Data Synthesis and Analysis

A thematic analysis was conducted, and extracted data were organized into the following pre-defined themes, which represent the potential susceptibility factors to be explored: (i) the biological mechanism of colibactin, (ii) the genomic landscape of CRC in Africa, and (iii) risk factors associated with colibactin-induced CRC in Africa. The summary of each research paper’s findings is shown in Table 1.

**Table 1:** Summary of the key findings of the included studies under pre-defined themes FA: Fanconi anemia, HR: homologous recombination, MMR: mismatch repair, pks: polyketide synthase, *E.Coli*: *Escherichia coli*, CRC: colorectal cancer, SBS88: single base substitution signature 88, *APC*: Adenomatous Polyposis Coli, TP53: tumor protein P53, SASP: senescence-associated secretory phenotype, NOD2: nucleotide-binding oligomerization domain-containing protein 2, ATG16L1: autophagy related 16 like 1, CoPEC: colibactin-producing *Escherichia coli*, *FUT2*: Fucosyltransferase 2, IBD: inflammatory bowel disease

Author(s)	Year	Title	Summary of Findings
Lan et al. [3]	2019	Prevalence of pks gene cluster and characteristics of Klebsiella pneumoniae induced bloodstream infections	This study focuses on colibactin induces profound genomic instability, a recognized cancer hallmark, through a direct mechanism of DNA alkylation that results in interstrand cross-links, ultimately leading to double-strand breaks and chromosomal rearrangements.
Bellido et al. [4]	2015	POLE and POLD1 mutations in 529 kindred with familial colorectal cancer and/or polyposis: review of reported cases and recommendations for genetic testing and surveillance	This research shows recent genomic analyses have identified a distinct mutational signature in colorectal cancer (CRC) tissues linked to colibactin-producing E. coli (CoPEC) exposure, suggesting a direct role in driving tumorigenesis through mutations in key genes like APC and TP53. Germline or somatic deficiencies in DNA crosslink repair pathways (e.g., FA, HR, MMR) increase susceptibility to colibactin by allowing its DNA lesions to be converted into fixed, oncogenic mutations rather than being properly repaired.
Hampel et al. [6]	2022	Hereditary colorectal cancer.	The article demonstrates CoPEC can subvert host immune surveillance by disrupting epithelial integrity and secreting immunomodulatory factors, fostering a state of persistent immune activation that is conducive to tumor development. The specific oncogenic impact of colibactin's mutational signature (SBS88) is determined by positive selection for driver mutations in key genes like APC and TP53, which accelerates tumorigenesis. The carriage and prevalence of pks+ strains are modified by environmental factors, with Western-style diets (high-fat, low-fiber) and regional practices (antibiotic use, food processing) directly linked to their increased abundance in human studies. The presence of pks+ E. coli enables direct genotoxic damage to host epithelial DNA through colibactin production, initiating carcinogenesis by inducing driver mutations while promoting local inflammation and cellular disruption.
Zarei et al. [7]	2022	Determination of virulence determinants of Escherichia coli strains isolated from patients with colorectal cancer compared to the healthy subjects	This research compared Escherichia coli strains isolated from colorectal cancer patients and healthy controls to determine their virulence traits. The key finding was that strains from cancer patients carried significantly more virulence determinants, suggesting a potential role for pathogenic E. coli in colorectal carcinogenesis and highlighting them as possible biomarkers or therapeutic targets.
Fentie et al. [11]	2018	Bacterial profile, antibiotic resistance pattern and associated factors among cancer patients at University of Gondar Hospital, Northwest Ethiopia.	The article showed host glycan genetics (e.g., FUT2 secretor status) influence mucosal niche availability for pks+ bacteria by altering mucin glycosylation, thereby modulating colonization stability and genotoxin exposure levels. And this has led to a significant raise in incidence of Colorectal cancer
Carlos et al. [12]	2010	Escherichia coli phylogenetic group determination and its application in the identification of the major animal source of fecal contamination	This study analyzed the phylogenetic distribution of Escherichia coli from humans and multiple animal hosts, showing that population structures cluster by feeding habits—omnivores (humans) versus herbivores. Key findings highlight that subgroup B23 was unique to humans, group B1 dominated herbivores, and the use of phylogenetic groups and genetic markers proved effective for tracing the major animal sources of fecal contamination in water systems
Jansen et al. [13]	2019	Novel candidates in early-onset familial colorectal cancer	This article highlighted that Colibactin exposure can trigger a senescence-associated secretory phenotype (SASP) in cells, resulting in a pro-tumorigenic microenvironment characterized by chronic inflammation and tissue remodeling that supports cancer progression. Genetic variants that compromise innate immunity, mucosal barrier integrity, or inflammatory regulation (e.g., in NOD2, ATG16L1) increase colibactin exposure duration and intensity, thereby elevating cancer risk. Chronic inflammation, whether from inflammatory bowel disease or common regional infections, generates a mucosa that is both genetically vulnerable and permissive to colonization by genotoxic bacteria, with evidence showing colibactin can synergize with inflammatory damage to accelerate tumor development.
Rossi et al. [14]	2017	A survey of the clinicopathological and molecular characteristics of patients with suspected Lynch syndrome in Latin America.	The pathogenic impact of pks+ E. coli is shaped by microbiome ecology, as biofilm formation and physical proximity to the crypt epithelium—governed by bacterial adhesins and co-occurring species—concentrate genotoxin exposure on stem cells. Dietary patterns high in processed meats and fats and low in fiber create a colonic environment that favors the expansion and mucosal adherence of pks+ E. coli, thereby increasing epithelial exposure to colibactin while depleting protective metabolites that maintain mucosal barrier integrity. An individual's genetic susceptibility in DNA repair and inflammatory response is the key risk factor for colibactin's oncogenic effects, though environmental toxins may potentially worsen the damage.
Dougherty and Jobin [15]	2021	Shining a light on Colibactin biology	There is a clinically significant synergism whereby inflammation, as seen in IBD, promotes colonization by CoPEC, and in turn, colibactin genotoxicity exacerbates tissue damage, creating a vicious cycle that markedly elevates colorectal cancer risk.
Thakur et al. [16]	2019	Unveiling the mutational mechanism of the bacterial genotoxin colibactin in colorectal cancer.	colibactin, a genotoxin from pks⁺ E. coli, induces DNA alkylation that generates interstrand crosslinks, replication fork stalling, and double-strand breaks. By mapping the specific mutational signature in host cells, the authors provide direct mechanistic evidence linking colibactin exposure to genomic instability and colorectal cancer risk. Uniquely, this work unveils the precise DNA lesions and mutational footprints of colibactin, offering the strongest proof to date of its carcinogenic role in humans.

Discussion

In Africa, CRC is becoming increasingly acknowledged as a significant non-communicable disease burden. Although the incidence of CRC in the continent was historically lower compared with high-income nations, epidemiological statistics over the past three decades have consistently shown an increase in both incidence and mortality. Numerous factors, including changes in population demographics, dietary habits, lifestyle choices, and microbial exposure, contributed to this increase. Due to its mutational imprint in human CRC, colibactin, a genotoxic chemical produced by specific *E. coli* strains harboring the pks genomic island, has gained interest as a microbial risk factor [11]. Colibactin is considered a likely microbiological cause of colorectal carcinogenesis, as it produces DNA crosslinks and distinctive mutational signatures that can be identified in tumors [12]. Although the epidemiology of colibactin-induced CRC in Africa is poorly characterized due to limited studies, as shown in this review, patterns of risk and burden can be delineated using data from food safety, dietary surveys, microbiome investigations, and cancer registries.

According to the Global Cancer Observatory (GLOBOCAN) 2022 version 1.1, new CRC cases in Africa were around 70,428. It also lists 46,087 deaths from the disease that year, putting the ASIR for CRC for both sexes at 8.4 per 100,000. There are significant subregional differences in incidence rates, however. Central Africa has the lowest ASIRs, whereas North and Southern Africa have the highest (Table 2) [2].

**Table 2:** Top African countries with the highest colorectal cancer incidence ASIR: age-standardised incidence rates

Country	ASIR (world), incidence (per 100,000), 2022	2022 New Cases	2022 Deaths
Mauritius	13.0	370	173
Libya	12.0	974	660
Algeria	11.3	7,747	4,380
Tunisia	11.0	1,839	953
Morocco	10.8	5,306	2,892
South Africa	10.5	3,873	2,462
Ethiopia	10.2	6,551	4,863
Kenya	9.6	3,091	2,116
Zimbabwe	10.1	773	524
Somalia	9.8	755	623

Due to late presentation, inadequate screening, and a shortage of treatment facilities, mortality-to-incidence ratios remain high throughout the continent [13]. Crucially, it is anticipated that by 2050, the incidence of CRC in Africa will have more than doubled as a result of aging, population expansion, and lifestyle changes if present risk levels persist [17]. Microbial cofactors, such as *E. coli*, that produce colibactin can have a discernible impact on the incidence of CRC due to these epidemiological tendencies [1].

Urban-rural disparities are evident in the distribution of CRC across Africa. Incidence rates are consistently higher in urban centers such as Gauteng and the Western Cape (South Africa), Cairo and Alexandria (Egypt), Abuja (Nigeria), and Casablanca (Morocco) than in rural regions [14]. This gradient likely reflects differences in healthcare access, antibiotic exposure, dietary habits, and sanitation factors that collectively shape the gut microbiome and influence colonization by colibactin-producing bacteria. Limited screening infrastructure in rural areas contributes to underdiagnosis, whereas higher antibiotic use and westernized diets in urban populations may disrupt microbial homeostasis, favoring colonization by pks+ *E. coli* strains that produce colibactin, a genotoxin that induces DNA alkylation and double-strand breaks. Moreover, variations in sanitation and wastewater management may increase exposure risks in densely populated settings by contaminating food or water. Age distribution also represents a notable epidemiologic distinction: Africa bears a disproportionately high burden of early-onset CRC (diagnosis before age 50), in contrast to high-income countries, where incidence peaks among older adults [8]. Genomic findings exhibiting the mutational signature of colibactin in younger patients confirm its participation in this pattern, indicating that early-life colonization by pks+ *E. coli* may predispose individuals to CRC many years later [12].

Colibactin-Producing E. coli and CRC

CRC is one of the leading causes of cancer-related morbidity and death globally [13]. It is a complex condition influenced by interactions between dietary factors and gut microbial communities, as well as genetic predispositions. A growing number of microbiological variables have been linked to colorectal carcinogenesis, including specific strains of *E.Coli*. Colibactin is a secondary metabolite produced by *E. coli* strains that carry the pks genomic island. It is a genotoxin that can cause DNA damage to host cells [14]. According to increasing evidence, colibactin-producing *E. coli* significantly contributes to the development and spread of CRC by promoting tumorigenesis, inflammation, and genetic instability. Trillions of bacteria reside in the human colon, forming a complex ecosystem that supports mucosal integrity, immunity, and digestion. Dysbiosis, or disturbances in this equilibrium, has been linked to CRC. Due to their ability to influence carcinogenic processes, certain bacteria, including enterotoxigenic *Bacteroides fragilis*, colibactin-producing *E. coli*, and *Fusobacterium nucleatum*, are classified as oncogenic microorganisms. Because of its direct potential to damage DNA, colibactin-producing *E. coli* has garnered particular attention among them.

**Figure 2:**
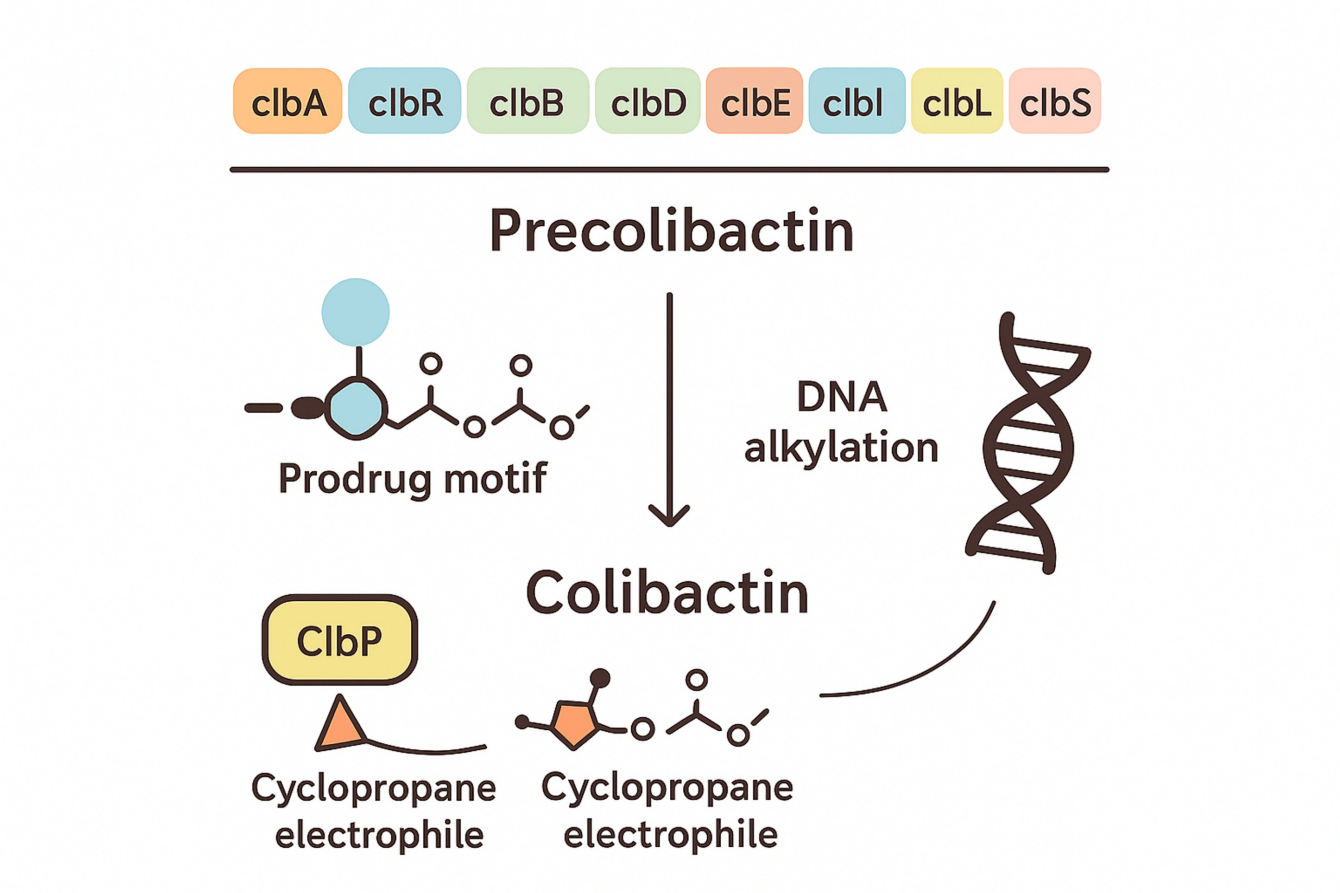
Biosynthesis and activation of colibactin The 54-kb pks genomic island encodes 19 genes organized into polycistronic and monocistronic units responsible for colibactin production. These include non-ribosomal peptide synthetase (NRPS), polyketide synthase (pks), and hybrid enzymes, along with accessory proteins. Expression is regulated by the transcriptional activator ClbR and the pantetheinyltransferase ClbA. The inactive precolibactin precursor is processed by ClbP, resulting in the active genotoxin featuring dual cyclopropane warheads that preferentially target adenine-rich DNA motifs [14]. Figure adapted from: Dougherty et al., [15]; licensed under CC BY 4.0 Attribution 4.0 International (https://creativecommons.org/licenses/by/4.0/)

Enzymes encoded in the pks genomic island, the 54-kb chromosomal cluster present in some *E. coli* strains, especially those belonging to the B2 phylogenetic group, produce the genotoxic substance colibactin. The hybrid system that this island encodes, known as non-ribosomal peptide synthetase-polyketide synthase (NRPS-PKS), is in charge of the manufacture of colibactin (Figure 2) [7,15].

Although the molecule is unstable and challenging to separate, biochemical research has demonstrated that colibactin possesses structural features that enable it to alkylate DNA, resulting in double-strand breaks and interstrand cross-links (ICLs). Colibactin-induced damage frequently results in senescence and genomic instability, which creates a mutagenic environment that promotes the development of cancer, in contrast to many toxins that cause apoptosis [7].

The Multifaceted Role of Colibactin in Colorectal Carcinogenesis

**Figure 3:**
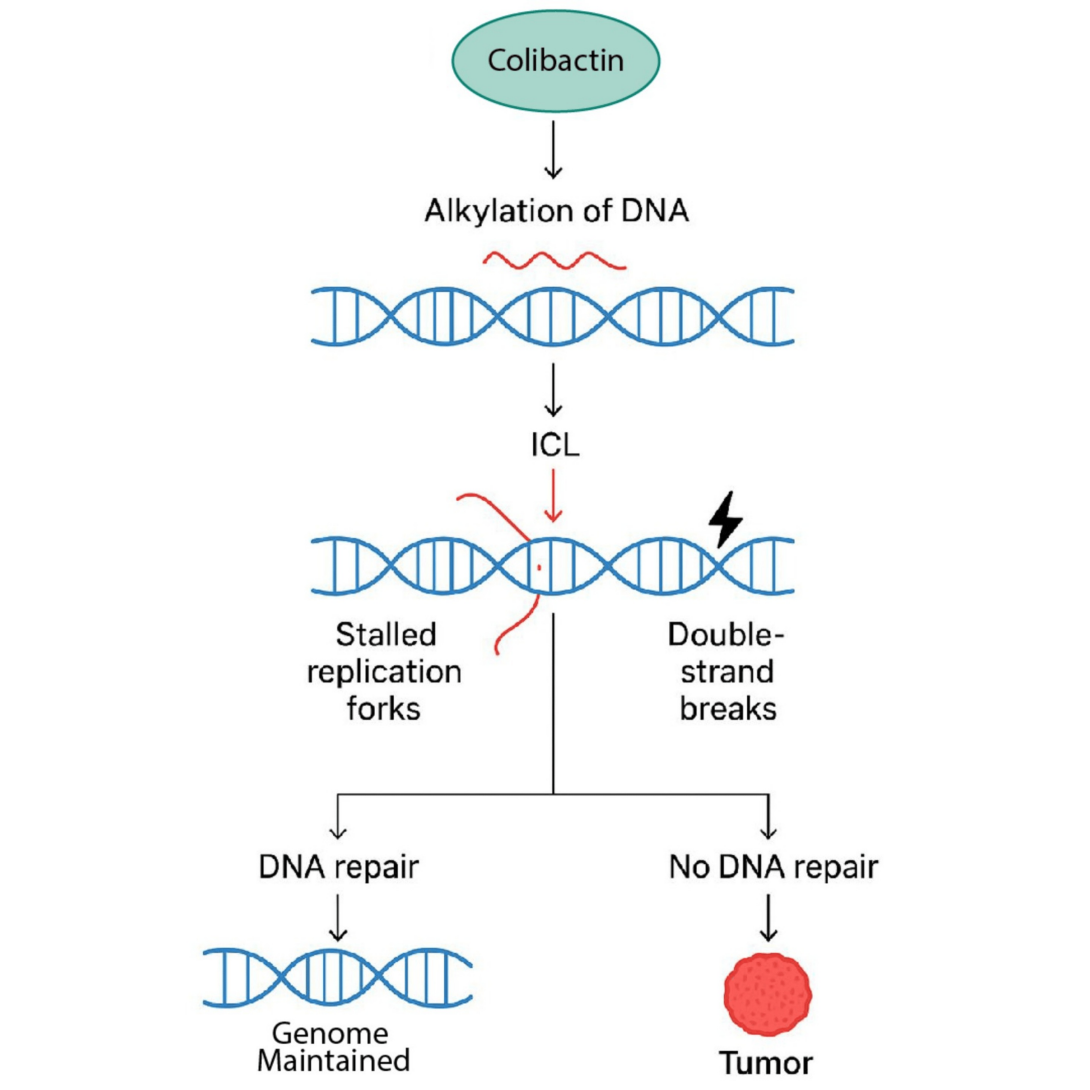
Colibactin influencing DNA damage Colibactin induces DNA alkylation with interstrand crosslinks and double-strand breaks; repair maintains genome integrity, absence of repair promotes tumorigenesis. ICL: interstrand cross-link Image Credit: Authors

The genotoxin colibactin, produced by pks+ *E. coli*, is a key microbial driver of CRC through a convergent mechanism that directly damages host DNA and alters the tumor microenvironment [4,6,12]. Its primary oncogenic action is through the alkylation of DNA, creating ICLs that stall replication forks and lead to double-strand breaks (Figure 3) and chromosomal instability, if not faithfully repaired [3,6,13,16].

This process leaves a distinct mutational signature, known as the single base substitution signature 88 (SBS88) or insertion/deletion signature 18 (ID18), in human tumors. It is capable of initiating mutations in critical driver genes, such as *APC* and Tumor Protein P53 (*TP53*) [4,7]. Beyond direct genotoxicity, colibactin promotes a pro-tumorigenic niche by inducing cellular senescence, known as the senescence-associated secretory phenotype (SASP), which increases inflammatory mediators and growth factors, and by compromising epithelial barrier function to enable persistent immune activation [6,13].

The risk of developing colibactin-induced CRC is not uniform but is instead determined by a complex interplay of environmental, microbial, and host genetic factors. This is particularly salient across the diverse populations of Africa. Key modifiable risk factors include diets high in processed meats and low in fiber, which elevate DNA-damaging bile acids and create a microbial niche favorable for pks+ *E. coli* proliferation and mucosal biofilm formation [5,13]. Dietary surveys across Africa reveal marked regional variations: populations in parts of West and East Africa maintain higher intakes of fiber and legumes, whereas urban centers in North and Southern Africa show increasing consumption of ultra-processed foods and animal fats. These nutritional transitions parallel the continent’s rising colorectal cancer incidence and underscore the need for targeted prevention strategies tailored to regional dietary patterns [18].

Furthermore, the high prevalence of chronic inflammatory co-infections (e.g., schistosomiasis, helminths, human immunodeficiency virus) synergizes powerfully with colibactin by damaging the mucosal barrier, facilitating bacterial colonization, and generating a microenvironment rich in DNA-damaging reactive species [13,14].

Ultimately, an individual's genetic susceptibility acts as the final arbiter of risk. Genetic variants that disrupt the repair of colibactin-induced ICLs, as in the Fanconi Anemia pathway (Complementation Group A or D2), homologous recombination (e.g. Breast Cancer Gene 1/2), or mismatch repair (e.g. Lynch syndrome, dramatically increase the likelihood that DNA damage will be fixed as oncogenic mutations [4,6,13,14]. Additionally, polymorphisms in genes governing innate immunity: Oligomerization Domain-Containing Protein 2 (NOD2), Toll-Like Receptor (TLRs), or autophagy such as Autophagy Related 16 Like 1 (ATG16L1), and mucosal integrity like Mucin 2 (MUC2), Fucosyltransferase 2 (FUT2) can increase susceptibility by weakening barrier defense and allowing for greater bacterial colonization and persistence [11,13,14].

Implications For Future Research and Public Health Initiatives

The growing burden of colibactin-associated CRC in Africa, driven by exposure to pks+ *E. coli* and influenced by genetic and environmental diversity, highlights the need for integrated, region-specific research. Key recommendations include: (i) linking host germline sequencing, tumor genomic profiling for colibactin-related mutational signatures, and microbiome analysis with functional organoid models that reflect African contexts, (ii) building local research infrastructure, surveillance capacity, and biobanks, (iii) prioritizing prevention through improved food and water safety, antibiotic stewardship, and nutrition, (iv) introducing risk-based screening and exploring microbiome-targeted therapies, and (v) ensuring sustained investment, ethical oversight, community participation, and collaboration across research and healthcare sectors for long-term impact.

## Conclusions

Colibactin-associated carcinogenesis is a multi-step process where environmental and lifestyle factors increase exposure to pks+ bacteria. At the same time, host genetics determines the efficiency of the immune and DNA-damage responses to that exposure. While there are limited studies in this population group, it is important to acknowledge that the ongoing epidemiological shifts in Africa, characterized by urbanization and dietary westernization, are likely amplifying this risk by increasing colonization with genotoxic strains in populations with a diverse and under-characterized genetic susceptibility profile. Therefore, targeted public health strategies and genetic research within African cohorts are critically needed to mitigate this growing burden.
